# Supplementary material for: Coupling Square Wave Anodic Stripping Voltammetry with Support Vector Regression to Detect the Concentration of Lead in Soil under the Interference of Copper Accurately
Source: Sensors (Basel). 2020 Nov 27;20(23):6792. doi: 10.3390/s20236792 (PMC7731166; doi:10.3390/s20236792)
Supplement: Supplementary file 1 [file sensors-20-06792-s001.pdf]

sensors

Supplementary Materials

Coupling square wave anodic stripping voltammetry with support vector regression to detect the concentration of lead in soil under the interference of copper accurately

Ning Liu [1,2], Guo Zhao [3], and Gang Liu [1,2]

[1] Key Laboratory of Modern Precision Agriculture System Integration Research, Ministry of Education of China, China Agricultural University, Beijing, 100083, China; ningliu@cau.edu.cn (N.L.).
[2] Key Laboratory of Agricultural information acquisition technology, Ministry of Agriculture and Rural Affairs of China, China Agricultural University, Beijing 100083, China; pac@cau.edu.cn (G.L.)
[3] College of Artificial Intelligence, Nanjing Agricultural University, Nanjing, 210031, China; zhaoguo@njau.edu.cn (G.Z.)
* Correspondence: pac@cau.edu.cn; Tel.: +86-1391-007-5680 (G.L.)

3.1. Electrochemical Activity of the Bare GCE

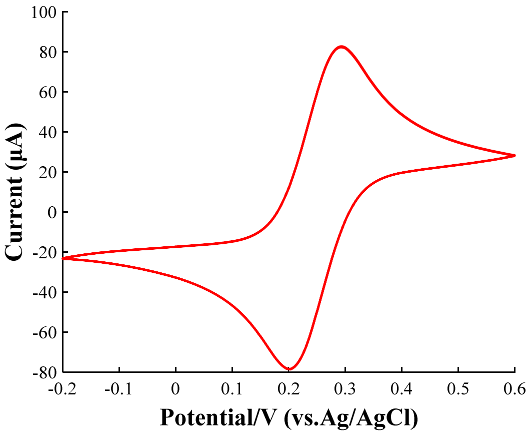

Figure S1. CV curve of the bare GCE in a mixture solution with 5 mM $[Fe(CN)_6]^{3-/4-}$ and 0.1 M KCl.

3.6.2 Analysis of the SVR Model Result for Pb(II) Concentration Detection

Table S1. Prediction results of the SVR model optimized by grid search for Pb(II) concentration.

Dataset	R^2	*RMSE* (μg/L)
training dataset	0.9954	0.9891
test dataset	0.9942	1.1204

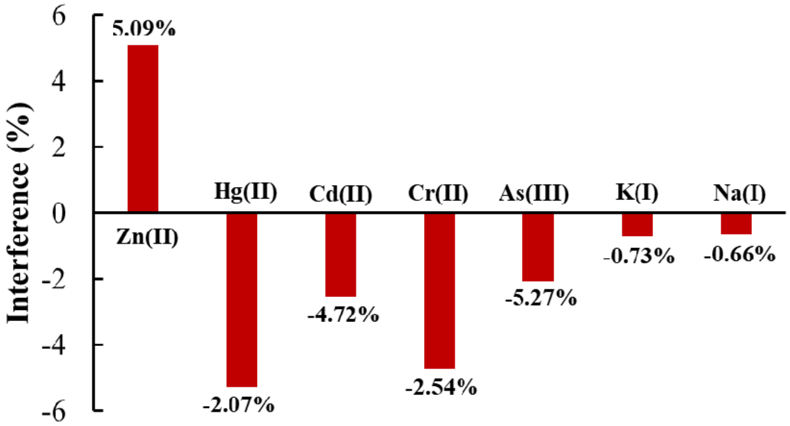

Figure S2. Interference of other cations on the stripping peak current of Pb(II) .
